# Supplementary material for: Are carbon emissions trading and green financial instruments synergistic? -Comprehensive quantitative research based on content analysis
Source: PLoS One. 2024 Mar 7;19(3):e0298601. doi: 10.1371/journal.pone.0298601 (PMC10919601; doi:10.1371/journal.pone.0298601)
Supplement: S1 Data — (ZIP) [file pone.0298601.s001.zip › Data/地级市Z_F工作报告.zip!/╔╜╬≈/┤≤═1⁄4╩╨/└┤╫╘╣1⁄2╓┌║┼í╛┬φ┐╦╩2╛▌═°í┐_2014.docx]

各位代表:

现在,我代表市人民政府,向大会作政府工作报告,请予审议,并请市政协各位委员提出意见。

一、2013年工作回顾

过去的一年,面对煤炭工业运行困难、经济下行压力增大、社会稳定矛盾突出的严峻形势,我们紧紧围绕“转型发展、绿色崛起”战略,按照“一三四十”总体部署,全力推进十大工程,直面矛盾,埋头苦干,争先进位,负重赶超,主要经济指标增幅在全省综合进位0.75,空气质量前进4位,国际太阳能竞赛成功举办,国家园林城市成功创建,国家新能源示范城市成功入围,十件实事圆满完成,各项工作稳中有进,稳中有为,稳中向好。

一年来,我们坚持把发展作为第一要务,多措并举稳增长。制定和落实“同煤同用”、煤电互保、银企对接等措施,加强产销衔接服务,促进实体经济发展。地方煤炭生产逆势增长57.8%,工业产品销售率提高1.6个百分点,增幅全省第一。金融机构贷款增加180亿元,增幅全省第一,存贷比提高3.85个百分点。“六位一体”推进重点工程建设,完成投资1185亿元。出台促进民营经济发展30条,民间投资增长50.3%。土地、资金等关键要素指标向县域经济倾斜,分配土地指标5811亩,增长180%;财政补助资金7.45亿元,增长31.28%,7县经济总量占全市比重提高0.6个百分点,公共财政预算收入比重提高1.52个百分点,固定资产投资比重提高6.1个百分点。粮食生产克服多重自然灾害影响,实现“四连增”,突破20亿斤。一系列举措保障了经济稳步增长,全市地区生产总值增长8.3%,规模以上工业增加值增长10%,固定资产投资增长24.6%,社会消费品零售总额增长14%,公共财政预算收入增长17.8%,城镇居民人均可支配收入增长10.1%,农民人均纯收入增长12.8%。除地区生产总值和工业增加值外,五项主要经济指标增速好于全省平均水平。居民消费价格指数控制在2.7%,城镇登记失业率控制在2.8%;市区二级以上良好天数全省第二,PM2.5年均浓度值全省最低,万元GDP综合能耗降幅全省第一,污染物排放实现控制目标,安全生产死亡人数低于省控目标。

我们坚持把综改作为工作统领,多点突破增活力。出台三年实施方案和年度行动计划。“五规合一”完成用地分类标准、技术规程、数据底板“三统一”。取消、下放审批事项32项,市级投资审批权全部下放各县区。严格实行“两集中、两到位”,审批、服务事项全部纳入电子监察系统。出台户籍制度改革意见和实施细则。适时调整车用燃气价格、出租车运营价格,实行了阶梯式水价。积极推动灵丘尾矿库用地管理、阳高农村土地承包经营权抵押、浑源林权质押等试点工作。建立政府性债务防控化解制度和风险预警机制。成立市金融工作机构,发挥市场作用,拓宽融资渠道,归还银行贷款本息36.2亿元,做到了政府工程有序还欠、按进度付款、不拖欠农民工工资,兑现了“五个凡是”的承诺,维护了全市大局和建设队伍稳定。

不断扩大开放。我市被列为全国性综合交通枢纽城市。实现航空口岸临时开放,新增恢复9条航线,大同至香港通航,机场旅客吞吐量增长27%,增幅全省第一。接待国内外游客2355万人次,旅游总收入增长23%。成功举办世界养生大会、云冈文化旅游节等重大文化活动,大同的美誉度和开放度不断提升。

我们坚持把转型作为主攻方向,多业并重调结构。以煤为基、多元发展,大力实施百企强市、百园立农工程。同煤集团跻身世界500强。全市新增火电装机66万千瓦、风电40万千瓦、光电20万千瓦,总装机达到870万千瓦,保持全省第一。煤炭产业链条不断延伸。同煤60万吨甲醇试生产、10万吨活性炭主体完工,同车10万吨活性炭开工建设。新成新材料成为全省唯一在新三板挂牌企业。新增山西名牌产品7件、山西著名商标14件。成功申报“恒山黄芪”为国家地理标志保护产品、“大同县黄花”为国家地理标志证明商标。建成重点农业园区69个,新增设施农业3.64万亩,发展膜下滴灌2.45万亩,实现脱贫4.6万人。阳高县成为国家养生(养老)示范基地,灵丘空中草原和阳高大嘴窑杏园入选中国美丽田园景观。万昌物流园区列入交通部重点扶持项目,42万平方米云中商贸物流园投入运营,40万平方米新发地物流园局部试运营。第三产业占比首超二产,经济结构更趋合理。

我们坚持把民生作为保障重点,多方努力促和谐。全市财政民生支出192.8亿元,增长25.3%,占公共财政预算支出达到82.4%。百校兴教工程深入推进,新增3个集团校,实行全市义务教育阶段新生同一天电脑编班,体育中考全部实行机考。大同一中南北校区、五中、十二中等5所市直学校投入使用。城乡安居工程统筹推进,开工城镇保障房2.06万套,完成农村危房改造2.08万户,完成农村住房抗震改建4000户。投资1.32亿元,安装农村路灯2.3万盏,120万农民告别了摸黑走路的历史。面对汛期强降雨的严峻形势,及时转移安置农村受灾群众8452户,紧急搬迁安置古城危房1832户。恒安新区、魏都新城等小区治安管理和物业服务逐步加强。收入倍增工程有序推进,城镇新增就业5.67万人,转移农村劳动力3万人。增加企业退休人员基本养老金90元/月,提高城乡居民基础养老金10元/月。分别提高城乡低保标准30元/月、24元/月。解决16家涉拆自收自支事业单位1833名职工的工资待遇、养老保障问题。平安创建工程扎实推进,设立500万元安全生产有奖举报专项资金。改革警务运行机制,探索综合警务执法,设立111个警务工作站,开展严打整治专项行动,遏制刑事案件高发势头,人民群众安全感明显增强。全运会金牌、奖牌、总分位居全省第一。

我们坚持把城镇化作为发展引擎,多元筹资搞建设。大力实施名城复兴工程。古城内环路全线贯通,和阳街、清远街改造完成,四牌楼、魁星楼、纯阳宫广场修复完工,代王府修复一期工程主体完成,南城墙征收拆迁全面完成,景观工程基本建成。新区建设持续推进,太阳宫建成投运,为国际太阳能竞赛提供了有力保障。博物馆开始陈列布展。图书馆、大剧院、体育中心外装修基本完成。大力实施城镇提质工程。城区列入住建部智慧城市(区)试点。建成城市道路62条97公里。开通浑源—广灵、王庄堡—大营2条高速公路90公里,实现了高速公路县县通。实施房屋征收1.6万户,拆除建筑150万平方米。浑源列为全省大县城建设试点。推广广灵县城建设经验,全市大县城建设完成投资148亿元,圆满完成“五个一”建设年度任务。城镇化率达到59.03%。大力实施生态建设工程。完成营造林32万亩,新增城市绿化面积177万平方米,新增集中供热面积800万平方米,实施供热计量改造140万平方米。御东污水处理厂达标验收,东、西郊污水处理厂扩建工程投入运行。浑源神溪湿地列入国家湿地公园建设试点。严格环保执法,对三户央企在同项目采取停产整顿措施,收到了企业受益、群众满意和环境改善的效果。将一名偷排污染物责任人移送司法机关,成为我市环境违法入刑第一例。大力实施城乡清洁工程。严格月考核、月通报制度,问责22人。市县两级投入3.5亿元,行政村全部配齐清扫保洁员、垃圾收集车,增加城乡环卫保洁人员1万人,为环卫工人发放早餐补助6元/天。清运积存垃圾90万吨,建设垃圾压缩站32座,垃圾焚烧发电厂日均新增处理量200吨,浑源、阳高垃圾处理场建成使用,城乡面貌大为改观。

我们坚持把依法行政作为基本准则,多管齐下抓落实。自觉接受人大法律监督和政协民主监督,办理人大代表建议111件、政协提案284件,办复率均为100%;提请审议地方性法规草案2件,制定政府规章1件。出台市政府工作1个规则、10个制度。严格工程建设管理制度,坚持政府项目依法合规。加强行政监察和审计监督,推行政务公开,接受群众监督,服务质量和发展环境逐步优化。把抓落实作为对干部品格、能力、作风、效能的考验,增强了干部的执行力,推动了各项工作的圆满完成。

这些成绩的取得,是省委、省政府和市委坚强领导的结果,是人大和政协监督支持的结果,是全市人民团结奋斗的结果。在此,我代表市人民政府,向全市人民、驻同解放军、武警官兵、公安民警和驻同单位,向各民主党派和人民团体,向所有关心支持参与大同改革发展的各界朋友,表示衷心的感谢和崇高的敬意!

我们也清醒地认识到,我市经济社会发展仍然存在一些差距和问题。突出表现在:经济总量不大、发展速度不快,GDP、规模以上工业增加值增速低于全省平均水平;一些重要指标在全省排位靠后,作为经济先导性指标的工业用电量增幅倒数第三,土地单位面积投资额倒数第一,建筑施工死亡人数超过省控目标;好项目数量少,大项目推进慢,雁同灌区等项目未能实施;城市低保人口和农村贫困人口占比高,居民收入增加难度大,群众生活改善任务重;行政效率不高,门难进,脸难看,话难听,事难办问题仍然突出,发展环境有待进一步改善。对于这些差距和问题,我们不回避、不容忍,要牢记责任使命,敢于担当,毫不懈怠,抓紧追赶,抓紧整改,决不辜负全市人民期盼。

二、2014年工作安排

2014年是综改攻坚年和项目见效年。今年政府工作的总体要求是:全面贯彻落实党的十八大和十八届三中全会、中央和全省经济工作会、全国和全省“两会”精神,坚持“稳中求进、改革创新”的总要求,围绕“转型发展、绿色崛起”战略,按照“一三四十”总体部署,以开展群众路线教育实践活动为契机,以十大工程为抓手,统筹兼顾,对标一流,争先进位,负重赶超,着力保持经济平稳较快增长,着力激发市场和社会活力,着力保障和改善民生,着力加强能力和作风建设,推动大同经济社会转型跨越发展。

综合分析各种因素,今年的发展形势比去年更加严峻复杂,煤炭、电力、服务业市场持续低迷,财政收支矛盾更加突出。必须进一步增强忧患意识,树立底线思维,抢抓发展机遇,做好各项工作。今年全市经济社会发展的主要目标为:

经济发展预期性指标:地区生产总值增长9%左右,规模以上工业增加值增长11%左右,固定资产投资增长20%,社会消费品零售总额增长14%左右,公共财政预算收入增长9%左右。

民生保障性指标:城镇居民人均可支配收入增长10%左右,农民人均纯收入增长11%以上,居民消费价格总水平涨幅控制在3.5%左右,城镇新增就业岗位5.6万个,城镇登记失业率控制在4.2%以内。

安全环保约束性指标:万元地区生产总值综合能耗下降3.5%,二氧化碳、二氧化硫、化学需氧量、氨氮、氮氧化物、烟尘、工业粉尘排放量和安全生产控制在省下达指标之内。

转型综改是山西改革发展的统领和切入点。十大工程体现了“五位一体”的总要求,是政府工作的总抓手。开展国家环保模范城市、新能源示范城市、卫生城市、智慧城市“四城联创”,关系民生、关系环境、关系转型。我们必须举全市之力加以推进,使经济健康发展,社会和谐稳定,民生持续改善,使大同天更蓝、地更绿、水更清、城更美。

(一)突出综改统领,深入实施重要领域、关键环节改革,推动改革创新取得新突破。

深化土地改革,提高利用效率,着力解决土地瓶颈制约问题。全面开展城乡建设用地增减挂钩试点,加快推进浑源、广灵露天采矿用地试点和左云等五县区矿业存量土地整合利用试点。积极推进大同县等重度盐碱地和阳高、天镇未利用地转为建设用地试点,争取开展南郊、新荣、灵丘等工矿废弃地复垦利用试点。全面开展农村土地承包经营权确权颁证试点,依法有序推进土地流转,扩大流转规模。严格落实建设用地集约节约利用制度。坚持最严格保护制度,切实守住560万亩耕地红线。

深化投融资体制改革,强化金融支撑,着力解决政府性债务重、企业融资难问题。建立政府性债务预算管理制度,通过盘活现有资产、引入社会资本、推进政府购买服务等方式,有效化解存量债务,严格防控债务风险。推进旅游门票收益证券化,探索设立创投基金、文化旅游发展基金、城镇建设私募基金,开展农村土地承包经营权及宅基地抵押担保贷款试点。创新金融产品,优化金融服务,支持民间资本依法设立各类金融机构,规范典当、投融资公司发展。打击逃废债行为,改善金融生态,吸引更多金融机构入驻大同,推动金融市场多元化发展。

深化科技体制改革,创优人才环境,着力解决创新乏力、人才不足问题。鼓励企业加大研发投入,支持同煤、同车、大齿、威奇达等国家和省级工程技术研究中心、企业技术中心、重点实验室建设。搭建科技投融资平台,发挥政府投入四两拨千斤的作用,引导金融机构支持科技型企业发展。支持大同大学发展,促进产学研结合和技术成果转化。发展职业技术教育,培育实用技术人才,加强实用技术应用。实施招才引智、技术创新两个1000万奖励政策,留住人才,吸引人才。

深化工商登记制度改革,降低注册门槛,着力解决企业少、就业难问题。推进公司注册资本实缴登记制向认缴登记制转变,取消最低注册资本限制,放宽市场主体经营场所登记条件。推进先证后照向先照后证转变,由企业年检制改为年报公示制。深化国有企业改革,发展混合所有制经济。最大限度地激发民间投资热情,释放社会创新活力。

深化户籍制度改革,推进“人的城镇化”,着力解决外来人员落户难、城镇化质量不高问题。认真落实我市户籍制度改革意见,有序放宽市区落户条件,全面放开县城和集镇落户条件,将有能力有意愿并长期在城镇务工经商的人员转为城镇居民,引导人口有序集聚,促进人口合理流动。实行居住证制度,创新流动人口服务管理,促进基本公共服务均等化。

深化开发区管理体制改革,发展园区经济,着力解决产业集聚难、项目落地难问题。发挥好大同经济技术开发区“国家级”金字招牌优势,完善规划,理顺体制,扩区提质,做大做强。实行开发区向县区开放,采取“飞地经济”模式,建立利益分享机制,调动县区、开发区和企业多方积极性。积极申报塔山、左云煤化工、阳高龙泉、装备制造等园区为省级开发区。鼓励有实力的企业托建托管开发园区。打造要素集约、资源集聚、创新集成、产业集群的发展平台。

深化行政区划改革,优化生产力布局,着力解决资源配置不合理问题。积极稳妥推进市区行政区划调整,推进中心城区的乡镇改街道和村改居。有序扩大县城和重点镇的区划范围。探索县政府驻地镇的管理模式。按照设镇条件和程序,推进撤乡建镇。

深化教育综合改革,提升教育质量,着力解决学校活力差、发展不均衡问题。继续推进“四个一批”,加强学校领导班子建设,积极探索学校去行政化。落实县域内中小学校长、教师交流机制,探索教师“局管校用”体制,促进师资均衡。严格实行公办义务教育学校划片、就近、免试招生和新生同一天电脑编班制度,促进生源均衡。认真做好农民工随迁子女义务教育工作。推广城乡之间、强弱之间联盟校办学模式,扩大优质教育资源,促进办学条件均衡,从源头上破解择校难题。

深化医药卫生体制改革,办好基层医院,着力解决看病难、看病贵、看病远问题。推进以三医院、五医院、同煤总院为龙头的医疗联合体建设。支持一医院升级三甲。完善市属公立医院与县区、社区基层医院对口帮扶制度,扩大优质医疗资源。深化县级公立医院改革,探索乡镇卫生院编制纳入县级医院统一管理,争取90%的病人在县域内就诊。鼓励社会办医,形成多元格局,适应不同需求。完善重特大疾病保险制度,城镇职工医保报销限额由28万元提高到45万元,城镇居民医保由11万元提高到40万元,新农合在保持15万元的基础上探索开展商业保险,有效遏制因病致贫、因病返贫。

深化对外开放,承接产业转移,着力解决开放不足、活力不够问题。紧紧抓住京津冀一体化发展战略机遇,以能源、资源、农副产品输出为载体,以文化、旅游、交通为纽带,充分发挥区位、资源、人文等优势,主动融入京津冀经济圈,参与区域分工协作,吸引央企和院校入驻大同,打造山西面向京津开放的桥头堡。建立大同—张家口—乌兰察布区域一体化合作平台,利用好北京—张家口联申冬奥会契机,促进文化旅游业发展。全力推进大张客专早日开工,完成大西客专建设任务。协调推进广灵—蔚县、灵丘—涞源出省高速公路建设,尽早打通全面向东开放通道。加快航空口岸正式开放,形成大开放格局。创新招商方式,用好招商平台,增强招商引资的有效性和精准度。

改革是时代的主旋律,改革是政府的硬任务。要通过全面深化改革挖潜力、激活力、放红利,通过全面深化改革稳增长、惠民生、促发展。

(二)突出发展转型,深入实施百企强市、百园立农工程,推动经济建设取得新进展。

深入实施百企强市工程。坚持以煤为基、多元发展,扎实开展“项目见效年”活动,聚焦发展煤炭、电力、煤化工、装备制造、生物医药、商贸物流等优势产业,促进产业结构转型升级。加快推进同煤金庄、东周窑两个千万吨矿井验收达产,高家窑、小梁沟等地方矿井早日投产,新增地方产能570万吨。开工建设同煤塔山二期2×66万千瓦机组项目,全力推进国电湖东2×100万千瓦机组等重大项目前期工作,继续争取大唐国际御东2×35万千瓦等低热值煤电力项目获批,积极支持二电厂等企业发展,尽快培育电力产业成为支柱产业。重点抓好同煤60万吨甲醇等项目投产达效,尽早开工40亿立方米煤制天然气和60万吨烯烃项目。推进同煤10万吨活性炭投产,同车10万吨活性炭试生产,打造全国最大的活性炭生产基地。加快同车30吨货运电力机车批量生产,打造中国北方轨道交通产业基地。实施同药战略,支持国药威奇达快速发展,扶持普德药业上市,打造全球最大的抗生素原料基地。全力支持大秦公司发展,加快东小城、东信城、雁北广场、亿丰世贸等项目建设,鼓励发展电子商务,做大做强商贸物流支柱产业。

扶持发展民营经济。全面落实民营经济发展30条,全面放开市场准入,全面创优发展环境,全力解决民营企业融资难、用地难、审批难、政策落实难、项目推进难问题,抓大、壮中、育小、扶微,培育一批销售收入超亿元的“小巨人”企业,新增小微企业2000户以上,中小企业增加值增长13%,让民营经济和中小微企业成为经济增长的“金钥匙”,创业就业的“聚宝盆”,富民惠民的“摇钱树”。

大力发展县域经济。继续加大土地、资金等要素向县区倾斜力度,加快建设新荣石墨深加工、灵丘有机农业及废弃矿山治理、阳高绿苑饮品、天镇中地良种奶牛等一批县区项目。强化县区在园区建设中的主体作用,推进一县一园建设,促进县区主导产业特色化、规模化、集群化。强化对县域经济发展考核奖惩力度,形成你追我赶、争先进位的发展局面,优化县域经济结构,扩大县域经济总量。

深入实施百园立农工程。按照“一村一品、一县一业”规划,重点打造浑源黄芪、大同黄花、阳高杏、天镇蔬菜、灵丘苦荞、广灵食用菌、左云马铃薯、新荣小杂粮、南郊都市农业等种植园区,新增设施农业3万亩。坚持以农载牧,以牧富民,实施养羊“五个一”计划,新增140万只,建设左云、浑源两个百万养羊县。重点建设南郊四方奶牛、灵丘佳农肉牛、新荣中粮生猪、浑源泰丰黄芪羊等养殖园区。大力推进农业产业化,壮大农业龙头企业,发展农业合作组织。推进阳高守口堡等水库建设,加快南郊、灵丘等5个国家级小农水重点县区建设,改善农业基础条件。

(三)突出文化传承,深入实施名城复兴、百校兴教工程,推动文化建设取得新进展。

深入实施名城复兴工程。加快古城保护与发展,完成代王府中轴线一、二期修复工程,实施富力城商业项目,完成征收搬迁2500户。加快展览馆平移、联通大楼搬迁、西城墙修复,推进西城墙、北城墙房屋征收,实施北城墙景观工程。完成黄花街城墙加固和云冈五华洞窟檐保护工程,组织云冈景区创建全国佛教文化和石窟艺术旅游产业知名品牌示范园区。加大“四名”保护力度,加强广灵六棱山、左云摩天岭等风景名胜区和新荣得胜堡、天镇新平堡、阳高镇边堡等名镇名村保护。编制完成文化产业发展规划,组建文化旅游集团,办好云冈文化旅游节。北魏398文化创意园局部营业,文化科技产业基地开园迎客。加强县区“三馆一院”建设,不断完善基层文化设施。

深入实施百校兴教工程。拓宽融资渠道,加大投资力度,开展义务教育县域均衡达标。城区要率先达标,其余县区要对照标准,补齐短板,限期达标。打造示范学校77所,新建标准化幼儿园29所。加强教师队伍建设,严格规范办学行为,严肃查处乱收费、乱办班、乱补课问题。继续实行中考体育测试机考制度。出台落实优惠政策,鼓励发展民办教育。办好人民满意教育。

(四)突出民生改善,深入实施收入倍增、平安创建工程,推动社会建设取得新进展。

深入实施收入倍增工程。鼓励以创业带动就业,重点做好高校毕业生、零就业家庭、农村剩余劳动力、就业困难群体的就业工作,转移农村劳动力3万人,培训城镇登记失业人员2万人,扶持创业3000人。加快实现城乡居民养老保险并轨和“五险统征”,发放“社保一卡通”161万张,企业退休人员基本养老金和农村五保供养补助标准提高10%,城乡低保标准分别提高25元/月、22元/月。稳定物价尤其蔬菜价格,完善社会救助和保障标准与物价上涨挂钩联动机制,确保低保人群不因物价上涨而降低生活水平。以百企千村产业扶贫工程为抓手,抢抓国家集中连片扶贫的历史性机遇,争政策、争项目、争资金,打好扶贫攻坚战。把增加贫困人口收入作为对县区考核的重要内容,整合扶贫资源,改进扶贫方式,实施精准扶贫。改造集中连片贫困地区农村公路154公里,抓好同煤集团天镇现代农业示范区等项目建设,完成移民搬迁8945人,力争使4.6万贫困人口脱贫。全力办好为农民办的五件实事。

深入实施平安创建工程。牢记“三个敬畏”,强化红线意识,落实法律法规,严格安全生产政府监管责任和企业主体责任。深入开展煤矿、危化、消防、道路交通、建筑施工、森林防火等十个重点领域安全生产专项检查,严格实行安全考核“一票否决”制度,依法加大安全事故查处力度,坚决杜绝重大事故、减少一般性事故。用最严格的监管、最严厉的处罚、最严肃的问责,保障人民群众饮食、用药安全。加强社会综合治理,完善社区基础设施,加快天网工程建设,健全治安防控体系,增强群众安全感。严厉打击各类非法生产、违法建设和违法犯罪活动,为人民群众创造平安和谐的社会环境。

(五)突出“四城联创”,深入实施生态建设、城乡清洁工程,推动生态文明建设取得新进展。

深入实施生态建设工程,创建国家环保模范城市。持续推进大气治理,加强大气环境质量联防联控,PM2.5下降4%。古城内全面禁燃原煤,补贴推广型煤。新增集中供热面积350万平方米。设立500万元环境污染举报奖励资金,加大环保执法力度,严肃查处违法行为。持续推进造林绿化,继续实施京津风沙源治理、退耕还林还草、通道绿化、村庄绿化,抓好武周山、采凉山绿化,新增营造林44万亩,建设大泉山—火山群百万亩百公里生态旅游长廊。推进御河两岸、梓家村森林公园、社区公园绿化,新增建成区绿化面积100万平方米。加强浑源神溪湿地、广灵湿地公园等生态保护。持续推进水环境治理,加快御河、十里河等河流生态环境综合治理。让人民群众呼吸新鲜空气、喝干净水。

深入实施城乡清洁工程,创建国家卫生城市。进一步完善城市环卫基础设施,运营餐厨垃圾处理厂,开工污泥、粪便处理厂,加快建设左云、新荣、天镇、大同县垃圾无害化处理填埋场,配齐乡镇垃圾转运车,建成行政村垃圾密闭收集点。全面启动创卫工作,对照标准,制定规划,扎实推进。以公厕建设、旱厕改造、乱点治理为重点,以城中村、老旧社区、城乡结合部整治为关键,广泛宣传,全民参与,强力推进市容环境、交通秩序、传染病防治等达标建设,不断提升市民素质和城市管理水平。为人民群众创造干净整洁的生活环境。

优化能源结构,创建国家新能源示范城市。抓好新能源生产示范,建设新型综合能源基地,推进浑源、新荣等40万千瓦风力发电,南郊、天镇、大同县等20万千瓦光伏发电和阳高3万千瓦生物质发电项目。抓好新能源应用示范,推进12万千瓦分布式风力发电、2万千瓦分布式光伏发电项目,新增太阳能灯1.5万盏。抓好节能减排示范,发挥塔山循环经济园区示范效应,推动和正公司粉煤灰、英吉思公司煤矸石等综合利用项目扩能提质。加强工业、建筑、交通和公共领域节能,新建建筑全面执行绿色建筑标准,节能标准执行率达到100%,新增新能源汽车80辆。推动能源结构新型化、清洁化、低碳化。

加快信息化进程,创建国家智慧城市。加强顶层设计,整合现有资源,实现共建共享。推进智慧城区建设,打造智慧城管、智慧社区、智慧教育、智慧旅游、智慧物流。推进4G网络应用,实现宽带提速,夯实信息化基础,促进信息消费。推进大同IT产业园区威厚和罗克佳华项目建设。建成OA政府办公系统和十大工程项目化管理信息系统,提高行政效率。让人民群众享受便捷高效的智慧服务。

(六)突出城乡统筹,深入实施城镇提质、城乡安居工程,推动新型城镇化建设取得新进展。

深入实施城镇提质工程。认真实施《国家新型城镇化规划(2014-2020年)》,加快大同都市区建设。完善道路交通体系,开工建设北环桥、开源桥和外环路等85条110公里城市道路。加快御东新区建设,博物馆开放展览,大剧院、图书馆、美术馆、体育中心外装修全面完成,内装修与功能使用、市场运作进度匹配进行,完成御东公共活动走廊建设。开工建设汽车客运东站。推进口泉区提质,完成专项规划编制,改造泉新路,实施南环西路西延、甘河生态治理和落里湾污水处理厂项目。重点抓好浑源县、灵丘东河南镇等省级示范大县城和重点镇建设。坚持“四个结合”,实施“四项工程”,着力改善农村人居环境。走出一条中心城市带动、示范县镇引领、城乡统筹、协调发展的新型城镇化路子。

深入实施城乡安居工程。加快棚户区改造和沉陷区治理,新开工建设保障房4.6万套,农村危房改造1.5万户。加强建筑市场监管,规范建筑市场秩序,提高建筑工程质量。鼓励企业、园区建设公租房,创新公租房社会化建设模式。实行廉租房和公租房并轨,扩大保障对象和范围。依法征收房屋7500户,竣工安置用房1.7万套,实现回迁1.3万户,让拆迁户早日住上自己的房子,实现人民群众的住房梦。

支持国防和军队建设,深入开展双拥工作,争创全国双拥模范城“八连冠”。加强应急管理、防灾减灾工作。支持工会、共青团、妇联等群团工作。发展妇女儿童、老龄、残疾人和红十字会等社会事业。有序实施单独二孩政策,人口自然增长率控制在6.5‰以内。开展全民健身活动。抓好统计、气象、地震、人防、科普、档案、史志、外事、侨务、对台、援疆、民族、宗教等工作。搞好第三次经济普查。做好第五届居委会、第十届村委会换届工作。

三、加强政府自身改革建设

全面深化改革,政府改革是关键;实施十大工程,政府建设是保障。

加快改革步伐,建设服务政府。按照转职能、转方式、转作风的要求,完成市县政府机构改革任务,整合卫生和计生部门机构职能,理顺食品药品监管体制,理顺质监、工商管理体制,实现群众办事方便点。整合房屋登记、林地登记、土地登记等职责,建立统一办理不动产登记机构,实现产权登记方便点。整合市县乡村四级医疗卫生信息和资源,建立上下联动、资源共享的医疗卫生平台,实现群众看病方便点。整合公安巡警、交警、消防、辅警等警务资源,完善综合警务运行机制,实现为民服务方便点。

引深管理创新,建设效能政府。加快推进“五规合一”,完成五项规划合编工作,以一张图纸、一套标准、一套流程、一套规章,建立多部门联动审批机制,着力解决土地、规划、环评不衔接,审批周期长,项目落地难问题。推进政府管理创新,坚持对标一流定目标,一事一表做计划,按月调度抓落实,活力曲线抓考核,优化考核体系,改进考核工作,强化横向到边、纵向到底、责任到人的项目化管理。巩固“两集中、两到位”成果,加快政务大厅体系建设,做到行政审批快一点。加快公共资源交易市场建设,做到资源配置快一点。加快人力资源市场建设,做到就业创业快一点。发挥12345服务快车道作用,做到为群众办事快一点。

坚持依法行政,建设廉洁政府。把政府工作全面纳入法治轨道,用法治思维和法治方式履行职责。自觉接受人大及其常委会监督,主动接受政协民主监督,全面接受人民监督。推行负面清单制度,规范政府审批行为。严格落实“政府约法三章”,严格控制机构人员编制,确保财政供养人员只减不增,确保三公经费只减不增。深入开展以为民务实清廉为主要内容的群众路线教育实践活动,集中“四风”整治,落实八项规定,厉行节约,反对浪费,政府带头过紧日子。全面落实政府系统党风廉政建设责任制,进一步加强行政监察和审计监督,深入推进反腐倡廉建设,坚决查处腐败案件,努力做到干部清正、政府清廉、人民满意。

各位代表,改革发展任务艰巨,宏伟蓝图催人奋进。让我们紧密团结在以习近平同志为总书记的党中央周围,在省委、省政府和市委的正确领导下,与全市人民一道,真抓实干,攻坚克难,万众一心,众志成城,为转型跨越发展、全面建成小康社会而努力奋斗!
